# Associations Among Workplace Bullying, Resilience, Insomnia Severity, and Subjective Wellbeing in Chinese Resident Doctors

**DOI:** 10.3389/fpsyt.2022.840945

**Published:** 2022-02-18

**Authors:** Shaojiong Zhou, Jia Chen, Han Lin, Ying Ye, Yu Xiao, Na Ouyang, Shaomei Pan, Siqi Feng, Meiling Xie, Bingxian Li

**Affiliations:** ^1^Department of Neurology, Shantou Central Hospital, Shantou, China; ^2^Shantou University Medical College, Shantou, China; ^3^The Clinical Hospital of Chengdu Brain Science Institute, MOE Key Lab for Neuroinformation, University of Electronic Science and Technology of China, Chengdu, China; ^4^The Fourth People's Hospital of Chengdu, Chengdu, China; ^5^Department of Head and Neck Surgery, Cancer Hospital of Shantou University Medical College, Shantou, China; ^6^Wenzhou Seventh People's Hospital, Wenzhou, China; ^7^Kunming Medical University, Kunming, China

**Keywords:** workplace bullying, insomnia, resilience, subjective wellbeing, Chinese resident doctor, questionnaire survey

## Abstract

**Background:**

Although workplace bullying is common among medical workers, its associations with insomnia severity and subjective wellbeing are still unclear. Our study aimed to investigate these associations among resident doctors who are more vulnerable to both workplace bullying and insomnia.

**Methods:**

We conducted a cross-sectional survey of 1,877 resident doctors from 12 hospitals across 7 administrative regions in China. Workplace bullying, resilience, insomnia severity, and subjective wellbeing were evaluated by the Negative Acts Questionnaire-Revised (NAQ-R), the Chinese version of the Connor-Davidson Resilience Scale-10-item (CD-RISC-10), the Insomnia Severity Index, and the Index of Wellbeing, respectively. Further, a logistic regression analysis was used to analyze factors associated with insomnia. In addition, structural equation modeling (SEM) was applied to examine the associations among workplace bullying, resilience, insomnia severity, and subjective wellbeing.

**Results:**

In the present study, the rates of workplace bullying and insomnia were 51.4 and 33.2%, respectively. Workplace bullying (OR = 1.056, *p* < 0.001) and poor resilience (OR = 0.957, *p* < 0.001) were the factors associated with insomnia after controlling the confounding variables. Further, SEM of the present study revealed a direct relationship between workplace bullying and subjective wellbeing (std-β = −0.223, *p* < 0.001). In addition, insomnia severity (std-β = −0.071, *p* < 0.001) and resilience (std-β = −0.092, *p* < 0.001) can individually or collectively (std-β = −0.008, *p* < 0.001) mediate the indirect associations between workplace bullying and subjective wellbeing. However, resilience was found to act as a moderator only in the direct association between workplace bullying and subjective wellbeing.

**Conclusions:**

Workplace bullying and poor resilience were the factors associated with insomnia. Further, greater resilience acted as a buffer in the direct association between workplace bullying and subjective wellbeing, whereas both insomnia severity and resilience were critical mediators in the indirect associations between them.

## Introduction

Sleep problems due to various causes are prevalent among resident doctors (1). Several previous studies have reported that nearly one in two medical students and resident doctors have poor quality of sleep which is strongly associated with workplace bullying (2, 3). Workplace bullying is common among trainees in many medical fields, including surgery (4), psychiatry (5), internal medicine, and family medicine (6). Resident doctors are often occupied with intensive work and study and hence are more vulnerable to workplace bullying and insomnia. This may worsen their wellbeing and performance during clinical tasks (7, 8). Insomnia is strongly associated with cardiovascular diseases (9) and deterioration in cognitive performance (10), memory (11), and emotional function (12, 13). A previous meta-analysis of 17 studies involving 115,988 participants in China showed that the pooled prevalence of insomnia was 15.0% (14), and younger adults were more likely to suffer from insomnia. Therefore, it is important to focus on the association between workplace bullying and insomnia disorders among young doctors in China.

Workplace bullying refers to situations in which people are repeatedly subjected to negative behavior or mistreatment by others in their organization of work (15). Previous studies have found that negative workplace behavior is an occupational hazard in the healthcare sector (16). The prevalence of bullying varies among studies depending on the research design and assessment methods. According to a study involving Australian surgeons in 2016, 47% of the surgeons were victims of workplace bullying, and 68% witnessed workplace bullying (4). In the US, 37% of junior doctors have experienced bullying during their training (15). But in China, workplace bullying among resident doctors is overlooked. Some studies have suggested that workplace bullying is closely related to insomnia and the association is significantly closer for individuals with the miR-146a GG genotype (3, 17, 18). However, few studies have focused on mediating and moderating variables as well as the mechanisms underlying the association between workplace bullying and insomnia.

Resilience is the ability to mentally and emotionally cope with a crisis and help individuals recover, maintain or improve their mental health when challenged by risk factors (19, 20). Previous studies have shown that resilient characteristics are associated with lower anxiety and depression levels as well as reduced risk of suicidal ideation (21). When facing workplace bullying, individuals with poor resilience are prone to stronger emotional dysregulation than those with greater resilience (22). However, there is a paucity of research evaluating the mediating and moderating effects of resilience among workplace bullying, insomnia severity, and subjective wellbeing.

Subjective wellbeing is a self-reported measure of wellbeing. A tripartite model of subjective wellbeing developed by Ed Diener describes how people experience the quality of their lives (23). It comprises both reflective cognitive judgments, such as the satisfaction of life, and emotional reactions to life in terms of positive emotions vs. negative emotions (24). Subjective wellbeing not only reflects concerns of people and feelings regarding their quality of life (25) but also is closely associated with many important mental health indicators, such as anxiety, depression (26, 27), and insomnia (28). Insomnia has been shown to be detrimental to wellbeing of an individual and thus alleviation of sleep disorders is an effective way to improve subjective wellbeing (29, 30). However, few studies have assessed the associations between workplace bullying and subjective wellbeing.

The present study aimed to explore the associations among workplace bullying, resilience, insomnia severity, and subjective wellbeing in Chinese resident doctors. The hypotheses were: ① workplace bullying and resilience were the factors associated with insomnia; ② insomnia severity might be a mediator in the association between workplace bullying and subjective wellbeing, and the presence of resilience might act as a moderator.

## Materials and Methods

### Recruitment Procedure

A cross-sectional study was conducted through an online survey from January to April 2021. The participants of this study were recruited via WeChat network platform. The sampling of resident doctors was conducted from 12 hospitals across 7 administrative regions in China using convenience sampling strategy.

All resident doctors in hospitals possessed their own WeChat groups. An applet “Questionnaire Star” with an anonymous questionnaire was sent to the WeChat groups to invite resident doctors to participate in the survey. At the start of the questionnaire, all resident doctors were informed of the purpose of the present study and were requested whether they had a previous diagnosis of mental illness, such as schizophrenia, bipolar disorder, depression, anxiety, and obsessive-compulsive disorder. The study enrolled resident doctors who spent at least 6 months in the standardized residency training program at hospital. Those who had a previous diagnosis of mental illness or spent less than 6 months in the residency program were excluded from the present study. The present study was approved by the Research Ethics Board of Shantou University Medical College.

A total of 2,154 resident doctors completed the survey, and a total of 1,877 valid questionnaires were collected, yielding an effective rate of 87.1%.

### Questionnaires

#### Demographic Characteristics

The collected general demographics of the resident doctors included their gender, age, marital status, education level, resident grade, specialty, salary per month, and number of night shifts per month.

#### Workplace Bullying

Globally, the Negative Acts Questionnaire-Revised (NAQ-R) is constantly used to evaluate the prevalence of workplace bullying (31). The NAQ-R contains 22 types of negative behaviors, such as physical injury, verbal aggression, and social exclusion. The participants were required to select how often they were bullied by others in the workplace. Scores of between 1 and 5 for each question represent “never,” “now and then,” “monthly,” “weekly,” and “daily.” The overall score ranged from a minimum of 22 to a maximum of 110, and the higher scores indicated greater experiences of workplace bullying. A person with a total NAQ-R score ≥ 33 is usually identified as a victim of workplace bullying (32). Further, the Cronbach's alpha of the NAQ-R, which indicates the internal consistency of scales, was found to be quite satisfactory at 0.95.

#### Resilience

In the present study, resilience was evaluated using the Connor-Davidson Resilience Scale-10-item (CD-RISC-10) (33, 34). The CD-RISC-10 had a high internal consistency (Cronbach's alpha > 0.85) (34–36). It captures the core features of resilience over the preceding month. Further, the CD-RISC-10 comprises a total of 10 items that measure resilience on a five-point Likert scale. The participants were asked to rate the extent of their agreement (0, never; 1, few; 2, sometimes; 3, often; and 4, all the time). The rating score ranged from 0 to 40, and a lower score indicated poor resilience (36).

#### Sleeping Problems Assessment

In order to assess the sleeping problems of resident doctors, the insomnia severity index (ISI) was used in the present study. It comprises of seven items assessing (a) sleep-onset severity, (b) the maintenance of sleep, (c) problems of early morning awakening, (d) satisfaction with current sleep pattern, (e) interference with daily functioning, (f) noticeability of impairment due to sleep problems, and (g) level of distress caused by sleep problems. The respondents were requested to rate each item on a five-point Likert scale (0, none; 1, mild; 2, moderate; 3, severe; and 4, extremely severe). Items were summed. An overall score >8 suggests the presence of insomnia, and higher scores indicated more severe sleeping problems (37). The internal consistency of scales is great (Cronbach's alpha = 0.86) (38).

#### Subjective Wellbeing

The subjective wellbeing has been widely assessed using the index of wellbeing. This scale is composed of the following two sections: ① an index of general affect; and ② a life satisfaction questionnaire. This scale contains nine items measuring subjective wellbeing on a seven-point Likert scale ranging from 1 (strongly disagree) to 7 (strongly agree). The overall subjective wellbeing index score is the weighted sum of the scores of the above two sections. Further, a lower score indicates poor subjective wellbeing. In a latest study, it has been reported that the internal consistency of this scale was ideal (Cronbach's alpha = 0.90) (39).

### Statistical Analysis

In the present study, Chi-squared tests were applied to perform comparisons of the categorical variables between the insomnia group and non-insomnia group. Student *t*-test and Mann-Whitney test were used to compare differences between two groups for the normally and non-normally distributed continuous variables, respectively. A binary logistic regression analysis was also used to analyze the factors associated with insomnia, using workplace bullying and resilience as explanatory variables and controlling for potential confounding factors. The correlation analyses of normally and non-normally distributed continuous variables were tested through Pearson and Spearman correlation analyses, respectively.

Structural equation modeling (SEM) was performed using Mplus 8.3 to conduct path tests and examine the mediating effects of insomnia severity between workplace bullying and subjective wellbeing, as well as to evaluate the moderating effects of resilience among workplace bullying, insomnia severity, and subjective wellbeing. The significance of the mediating and moderating effects was examined using the bootstrap method. The 95% confidence intervals (95% CIs) were calculated using percentile and bias-corrected bootstrap methods with 5,000 iterations. Further, the mediating and moderating effects were significant if the 95% CI did not include a zero. The statistical significance of difference was set at *P* < 0.05. In addition, a simple slope analysis was performed to depict the moderating effects of resilience.

## Results

### Characteristics of Participants

The demographic characteristics of the resident doctors were displayed in Table 1. The mean age of the 1,877 resident doctors was 24.65 ± 1.47 years, 61.8% were female, 55.8% were single, 37.1% were in a relationship, and 7.1% were married. Further, the resident doctors in the first, second, and third years accounted for 39, 36.4, and 24.6% of all participants, respectively. In addition, among all resident doctors, 49.4% specialized in internal medicine, 22.3% specialized in surgery, 7.2% specialized in gynecology, and 16.2% specialized in other specialties. A total of 623 resident doctors had varying degrees of insomnia, yielding an insomnia rate of 33.2%.

**Table 1:** Characteristics of the resident doctors in the insomnia group and non-insomnia group.

	**Total (*n* = 1,877)**	**Non-insomnia (*n* = 1,254)**	**Insomnia (*n* = 623)**	***P*-values**
Gender, % (*n*)				0.001^**^
Male	38.2 (717)	35.6 (446)	43.5 (271)	
Female	61.8 (1,160)	64.4 (808)	56.5 (352)	
Age, years (*M* ±*SD*)	24.65 ± 1.471	24.596 ± 1.457	24.767 ± 1.496	0.017^**^
Marital status, % (*n*)				0.46
Single/Divorced	55.8 (1047)	56.8 (712)	53.8 (335)	
Relationship	37.1 (697)	36.4 (456)	38.7 (241)	
Married	7.1 (133)	6.8 (86)	7.5 (47)	
Education level, % (*n*)				0.638
Junior college	1.3 (24)	1.1 (14)	1.6 (10)	
Bachelor	34.5 (647)	34.2 (429)	35 (218)	
Master	63.7 (1,196)	64 (803)	63.1 (393)	
Ph.D.	0.5 (10)	0.7 (8)	0.3 (2)	
Resident grade, % (*n*)				0.001^**^
First-year	39 (732)	41.6 (521)	33.9 (211)	
Second-year	36.4 (683)	36 (452)	37.1 (231)	
Third-year	24.6 (462)	22.4 (281)	29 (181)	
Specialty, % (*n*)				0.209
Internal medicine	49.4 (927)	50.1 (628)	48 (299)	
Surgery	22.3 (419)	21.6 (271)	23.7 (148)	
Gynecology	7.2 (136)	7.8 (98)	6.1(38)	
Pediatrics	4.9 (92)	5.3 (66)	4.2 (26)	
Others	16.2 (303)	15.2 (191)	18 (112)	
Salary per month, % (*n*)				0.002^**^
<1,000 yuan	28.5 (536)	26.6 (334)	32.4 (202)	
1,001–3,000 yuan	39.2 (735)	40.8 (512)	35.8 (223)	
3,001–5,000 yuan	18.5 (347)	17.4 (218)	20.7 (129)	
>5,000 yuan	13.8 (259)	15.2 (190)	11.1 (69)	
Number of night shifts per month, % (*n*)				0.006^**^
0	10.8 (202)	11.9 (149)	8.5 (53)	
1–2	17.8 (335)	18.7 (235)	16.1 (100)	
3–5	51.9 (974)	51.7 (648)	52.3 (326)	
>5	19.5 (366)	17.7 (222)	23.1 (144)	
Workplace bullying, M ± SD	36.02 ± 12.969	32.84 ± 10.047	42.4 ± 15.581	0.000^**^
Person-related bullying	13.32 ± 5.169	12.09 ± 3.889	15.77 ± 6.409	0.000^**^
Work-related bullying	13.76 ± 5.236	12.56 ± 3.829	16.17 ± 6.666	0.000^**^
Organizational injustice	8.94 ± 3.834	8.18 ± 3.49	10.46 ± 4.041	0.000^**^
Resilience	23.24 ± 9.267	24.76 ± 9.486	20.19 ± 7.984	0.000^**^

*M ± SD, mean ± standard deviation*.

** *p < 0.01*.

*All bold values are statistically significant (p < 0.05)*.

Results of analyses of the difference between insomnia and non-insomnia groups revealed that males (*p* = 0.001), higher age (*p* = 0.017), higher resident grade (*p* = 0.001), lower salary (*p* = 0.002), more night shifts (*p* = 0.006), greater experience of workplace bullying (*p* < 0.001), and poor resilience (*p* < 0.001) were more significant in insomnia group.

### The Factors Associated With Insomnia

Results of binary logistic regression analyses were shown in Table 2. The males (OR = 1.251, *p* = 0.043) and status of a senior resident doctor (second-year vs. first-year: OR = 1.23, *p* = 0.123; third-year vs. first-year: OR = 1.588, *p* = 0.004) were both more likely to suffer from insomnia. Notably, resident doctors who had greater experience of workplace bullying (OR = 1.056, *p* < 0.001) and poor resilience (OR = 0.957, *p* < 0.001) were identified as the factors associated with insomnia after controlling for the confounding variables (gender, age, resident grade, salary, and number of night shifts).

**Table 2:** Binary logistic regression analysis of insomnia.

	**β**	**OR**	**95%CI**	***P*-values**
Male	0.224	1.251	(1.007, 1.553)	0.043^*^
Age	0.034	1.035	(0.951, 1.126)	0.425
Resident grade				0.017^*^
First-year (Reference)	/	1	/	**/**
Second-year	0.207	1.23	(0.945, 1.601)	0.123
Third-year	0.463	1.588	(1.157, 2.18)	0.004^**^
Salary per month				0.364
<1,000 yuan (Reference)	/	1	/	**/**
1,001–3,000 yuan	−0.101	0.904	(0.697, 1.172)	0.444
3,001–5,000 yuan	0.083	1.086	(0.796, 1.483)	0.602
>5,000 yuan	−0.228	0.796	(0.556, 1.139)	0.212
Number of night shifts per month				0.602
0 (Reference)	/	1	/	**/**
1–2	0.109	1.115	(0.734, 1.694)	0.61
3–5	0.201	1.223	(0.848, 1.764)	0.282
>5	0.259	1.296	(0.856, 1.962)	0.22
Workplace bullying	0.055	1.056	(1.046, 1.066)	0.000^**^
Resilience	−0.044	0.957	(0.946, 0.968)	0.000^**^

*Gender, age, resident grade, salary, and number of night shifts were controlled for in the analyses of workplace bullying and resilience*.

* *p < 0.05*;

** *p < 0.01*.

*All bold values are statistically significant (p < 0.05)*.

### Correlations of Variables

The results of the correlation analyses in the current study showed that all variables were correlated (Table 3). Expectedly, insomnia severity was positively associated with workplace bullying but negatively associated with resilience. Further, subjective wellbeing was negatively associated with both workplace bullying and insomnia severity but positively associated with resilience.

**Table 3:** Correlation analyses of all variables.

		**1**	**2**	**3**	**4**	**5**	**6**	**7**	**8**	**9**
1	Workplace bullying	1								
2	Person-related bullying	0.928^**^	1							
3	Work-related bullying	0.924^**^	0.855^**^	1						
4	Organizational injustice	0.869^**^	0.693^**^	0.686^**^	1					
5	Resilience	−0.302^**^	−0.290^**^	−0.278^**^	−0.244^**^	1				
6	Insomnia severity	0.410^**^	0.403^**^	0.378^**^	0.388^**^	−0.330^**^	1			
7	Subjective wellbeing	−0.437^**^	−0.383^**^	−0.387^**^	−0.423^**^	0.547^**^	−0.409^**^	1		
8	General affect	−0.425^**^	−0.372^**^	−0.373^**^	−0.415^**^	0.549^**^	−0.402^**^	0.962^**^	1	
9	Life satisfaction	−0.417^**^	−0.365^**^	−0.371^**^	−0.402^**^	0.500^**^	−0.389^**^	0.947^**^	0.834^**^	1

** *p < 0.01*.

### Structural Equation Model

**Figure 1:**
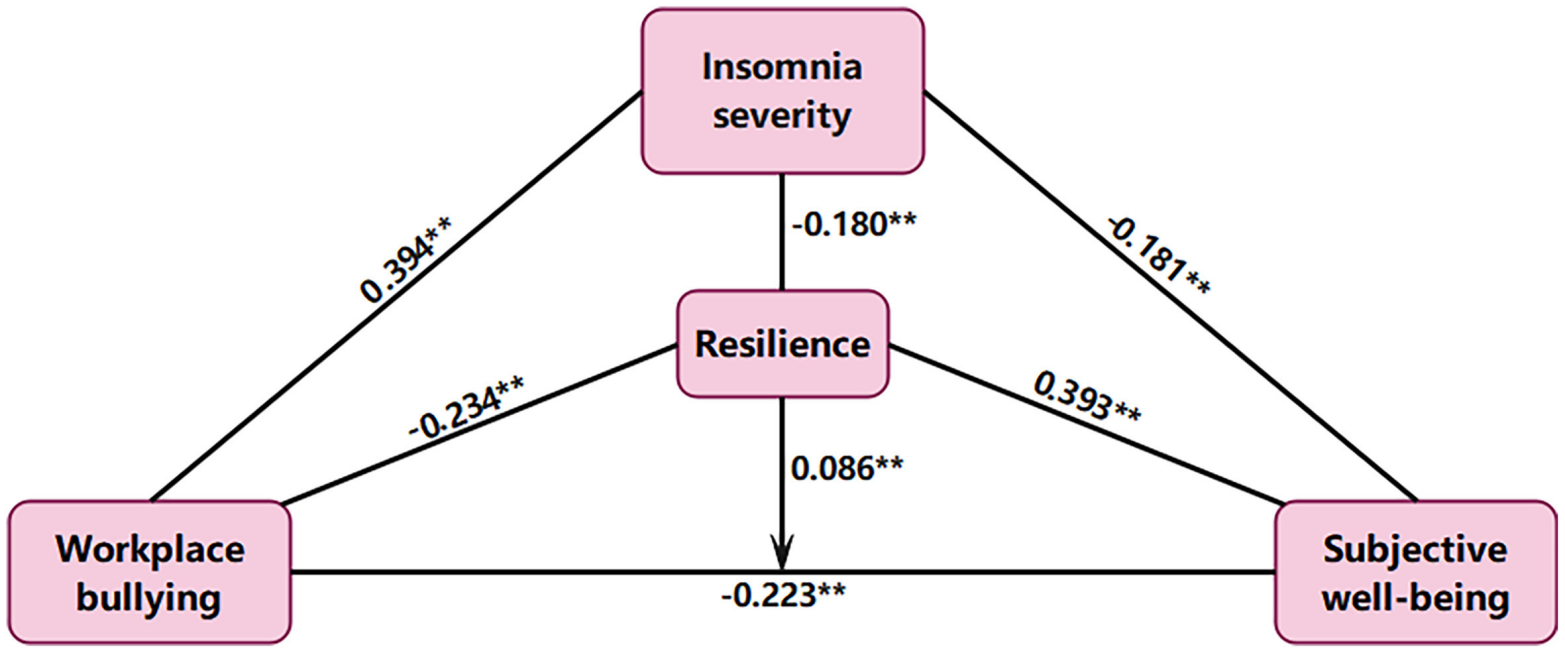
Structural equation model describing the indirect associations between workplace bullying and subjective wellbeing via insomnia severity and resilience. The direct association between workplace bullying and subjective wellbeing moderated by resilience is also shown. Standardized β are reported. ^**^p < 0.01.

The SEM was performed in the present study to test the study hypotheses (Figure 1). The indices of the model fit were ideal (SRMR = 0.008, CFI = 0.998, TLI = 0.986, RMSEA = 0.035, and RMSEA *p*-value < 0.05). In addition, the tests of single paths were all statistically significant (*p* < 0.001) and their coefficients [standardized β (std-β)] were displayed in Figure 1 and Table 4.

**Table 4:** Tests of single path.

**Single path**	**Std-β**	**S.E**.	***P*-values**
Workplace bullying → Insomnia severity	0.394	0.026	0.000^**^
Workplace bullying → Resilience	−0.234	0.025	0.000^**^
Workplace bullying → Subjective wellbeing	−0.223	0.024	0.000^**^
Insomnia severity → Subjective wellbeing	−0.181	0.025	0.000^**^
Resilience → Subjective wellbeing	0.393	0.024	0.000^**^
Resilience → Insomnia severity	−0.180	0.022	0.000^**^
Workplace bullying × Resilience → Subjective wellbeing	0.086	0.020	0.000^**^

*Std-β, standardized β; S.E., standard error*.

** *p < 0.01*.

*All bold values are statistically significant (p < 0.05)*.

According to the bootstrap results of the current study, a direct association was found between workplace bullying and subjective wellbeing (std-β = −0.223, S.E. = 0.024, *p* < 0.001). This indicates a direct effect between workplace bullying and subjective wellbeing. Further, the moderating effects of resilience were identified only on this direct association (std-β = 0.086, S.E. = 0.020, *p* < 0.001). Therefore, greater resilience would attenuate this direct association. In addition, results of the present study identified insomnia severity as a significant mediator between workplace bullying and subjective wellbeing. Besides, the bootstrap results showed that resilience was also a mediator among workplace bullying, insomnia severity, and subjective wellbeing.

### Mediation Analysis

A detailed results of bootstrap in the present study was as presented in Table 5. With an exception of for the direct effect between workplace bullying and subjective wellbeing [Path 1, std-β = −0.223, S.E. = 0.024, percentile 95% CI (−0.270, −0.177), *p* < 0.001], the rest four other indirect paths were also confirmed. Specifically, and in consistence with the hypotheses of the current study, insomnia severity was identified as a significant mediator between workplace bullying and subjective wellbeing [Path 2, std-β = −0.071, S.E. = 0.011, percentile 95% CI (−0.093, −0.050); *p* < 0.001]. Moreover, resilience had significant mediating effects on the association between workplace bullying and insomnia severity [Path 3, std-β = 0.042, S.E. = 0.007, percentile 95% CI (0.029, 0.057), *p* < 0.001], as well as on the association between workplace bullying and subjective wellbeing [Path 4, std-β = −0.092, S.E. = 0.012, percentile 95% CI (−0.117, −0.069), *p* < 0.001]. Besides, both resilience and insomnia severity mediated the relationship between workplace bullying and subjective wellbeing [Path 5, std-β = −0.008, S.E. = 0.002, percentile 95% CI (−0.011, −0.005), *p* < 0.001].

**Table 5:** Bootstrap results of direct and indirect effects between workplace bullying and subjective wellbeing.

**Effect and path**	**Std-β**	**S.E**.	**Percentile 95%CI**			**Bias-corrected 95%CI**		
			**Lower**	**Upper**	***P*-value**	**Lower**	**Upper**	***P*-value**
**Direct effect**								
Path 1	−0.223	0.024	−0.270	−0.177	0.000^**^	−0.269	−0.175	0.000^**^
**Indirect effect**								
Path 2	−0.071	0.011	−0.093	−0.050	0.000^**^	−0.095	−0.051	0.000^**^
Path 3	0.042	0.007	0.029	0.057	0.000^**^	0.029	0.058	0.000^**^
Path 4	−0.092	0.012	−0.117	−0.069	0.000^**^	−0.117	−0.069	0.000^**^
Path 5	−0.008	0.002	−0.011	−0.005	0.000^**^	−0.011	−0.005	0.000^**^

*Path 1, workplace bullying → subjective wellbeing; Path 2, workplace bullying → insomnia severity → subjective wellbeing; Path 3, workplace bullying → resilience → insomnia severity; Path 4, workplace bullying → resilience → subjective wellbeing; Path 5, workplace bullying → resilience → insomnia severity → subjective wellbeing; Std-β, standardized β; S.E., standard error*.

** *p < 0.01*.

*All bold values are statistically significant (p < 0.05)*.

### Moderation Analysis

Results of the current study indicated that the direct association between workplace bullying and subjective wellbeing was statistically significant. In addition, resilience was identified as a moderator only on this direct association (std-β = 0.086, S.E. = 0.020, *p* < 0.001). Based on the described findings, a moderation model was tested to obtain more details and to determine whether the direct association between workplace bullying and subjective wellbeing varied as a function of resilience.

The results of bootstrap on the moderating effects of resilience were demonstrated in Table 6. The coefficients of the moderating effects of resilience were all negative at high, medium, and low levels of resilience [High resilience: β = −0.029, S.E. = 0.006, percentile 95% CI (−0.042, −0.017); Medium resilience: β = −0.044, S.E. = 0.005, percentile 95% CI (−0.054, −0.035); low resilience: β = −0.059, S.E. = 0.006, percentile 95% CI (−0.070, −0.048)]. However, the pairwise comparison among the three levels of resilience were all positive and statistically significant [High-Low: β = 0.029, S.E. =0.007, percentile 95% CI (0.016, 0.045); High-Mean: β = 0.015, S.E. = 0.004, percentile 95% CI (0.008, 0.022); Mean-Low: β = 0.015, S.E. = 0.004, percentile 95% CI (0.008, 0.022)].

**Table 6:** Bootstrap results of the moderating effects of resilience on the direct association between workplace bullying and subjective wellbeing.

	**β**	**S.E**.	**Percentile 95%CI**			**Bias-corrected 95%CI**		
			**Lower**	**Upper**	***P*-value**	**Lower**	**Upper**	***P*-value**
High resilience	−0.029	0.006	−0.042	−0.017	0.000^**^	−0.042	−0.017	0.000^**^
Mean resilience	−0.044	0.005	−0.054	−0.035	0.000^**^	−0.054	−0.035	0.000^**^
Low resilience	−0.059	0.006	−0.070	−0.048	0.000^**^	−0.070	−0.047	0.000^**^
High-Low	0.029	0.007	0.016	0.045	0.000^**^	0.016	0.045	0.000^**^
High-Mean	0.015	0.004	0.008	0.022	0.000^**^	0.008	0.022	0.000^**^
Mean-Low	0.015	0.004	0.008	0.022	0.000^**^	0.008	0.022	0.000^**^

*High = mean + standard deviation; Low = mean – SD; S.E., standard error*.

** *p < 0.01*.

*All bold values are statistically significant (p < 0.05)*.

**Figure 2:**
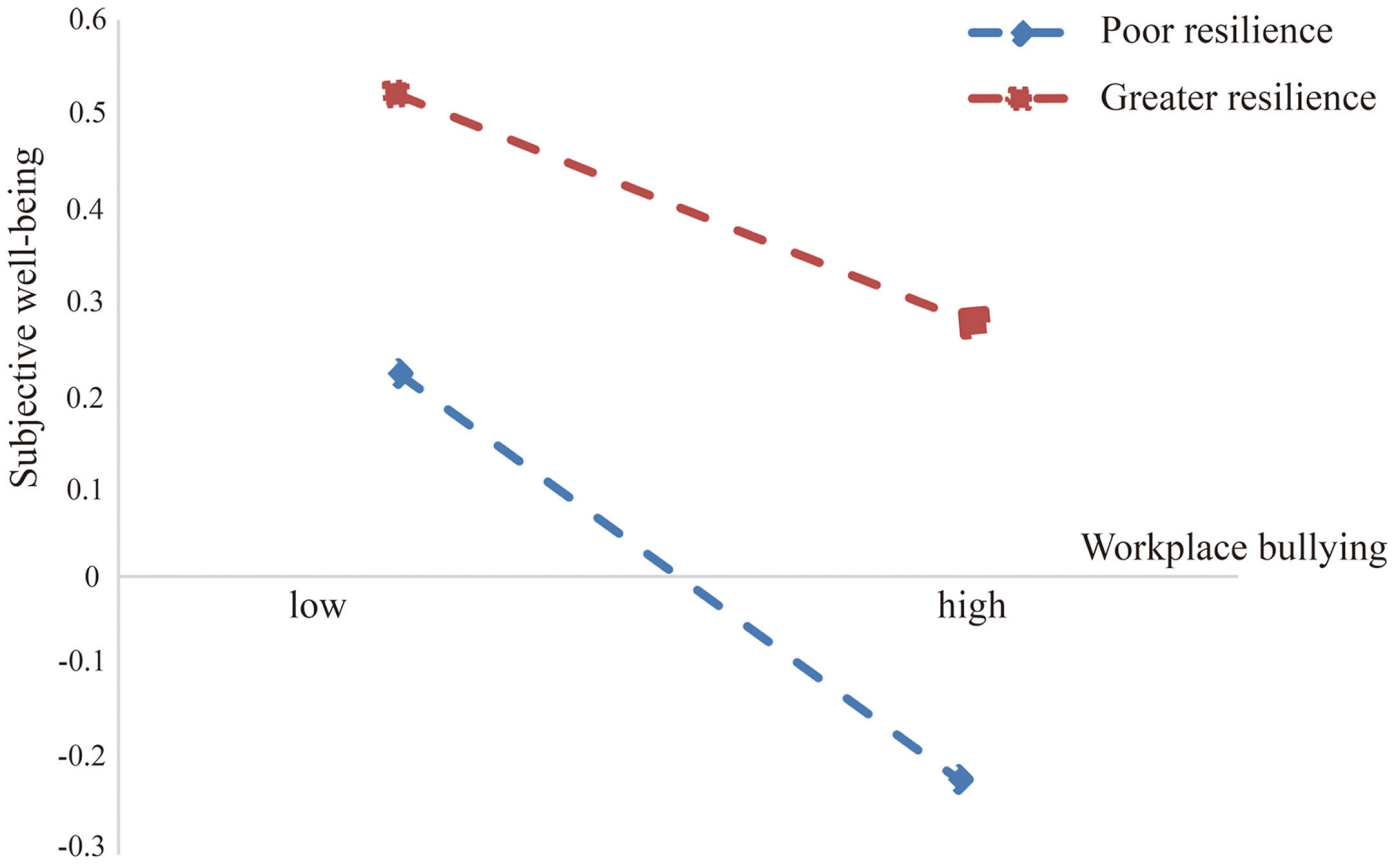
Simple slope analysis of the moderating effect of resilience.

Thus, the moderating effects of resilience on the direct association between workplace bullying and subjective wellbeing were significantly different among the three levels of resilience. Poor resilience would strengthen the direct association between workplace bullying and subjective wellbeing. Furthermore, simple slope analysis of the moderating effects of resilience, presented in Figure 2, showed that when exposed to equal levels of workplace bullying, the resident doctors with poor resilience had poor subjective wellbeing. Meanwhile, greater resilience would attenuate direct association between workplace bullying and subjective wellbeing.

## Discussion

Insomnia is a common disorder and according to a meta-analysis involving 17 studies with 115,988 participants, the pooled prevalence of insomnia in China has been reported to be 15.0% (14). However, the present study reported a much higher rate of insomnia (33.2%) among Chinese resident doctors, which is statistically similar to a study involving Chinese healthcare workers during the COVID-19 epidemic (38.4%) (40).

Previous meta-analysis revealed that at least 15% of workers experience workplace bullying (41). However, in the current study, the prevalence of workplace bullying among Chinese resident doctors was 51.4%, which is as high as the rate in Australia (47%) (4, 41). In addition, reports of previous studies have identified a much higher rate of workplace bullying in the healthcare sector, particularly among resident doctors. The reported rates of workplace bullying among resident doctors range from 30% in Ireland to 89% in India (8).

Further, workplace bullying and poor resilience were the factors associated with insomnia and the SEM of the study revealed close relationships among workplace bullying, resilience, and insomnia severity. Workplace bullying is a considerable environmental stressor that can increase sleep problems. Therefore, individuals with experiences of workplace bullying are more likely to suffer from insomnia (42–44) and have a higher use of sleep-inducing drugs and sedatives (45). Results of a recent meta-analysis showed that bullied workers have 2.13 higher odds of sleep problems than non-bullied workers (3). Further, inflammation may have effects on stress-induced insomnia among people exposed to workplace bullying (17).

Currently, there is limited research on mediating and moderating variables as well as mechanisms underlying the association between workplace bullying and insomnia severity. The results of the present study found that resilience, as a mediator, played a critical role in the association between workplace bullying and insomnia severity. This suggested that resilience may have a significant mediating effect rather than a moderating effect in the relationship between workplace bullying and insomnia severity.

Similarly, a study by Hansen found that workplace bullying is related to development of subsequent sleep problems and although physical activity has been shown to be beneficial for resilience, the association is not moderated by leisure-time physical activity (46–48).

In the present study, males had a higher rate of insomnia than females (37.8 vs. 30.3%), indicating that males and senior residence status were more likely to develop insomnia. The noted gender difference in the present study is different from those reported in previous studies. The rate of insomnia in females has been approximated to be 1.5 times higher than that in males (49). However, several studies have found that females have a better quality of objective sleep (50, 51). The studies highlighted that females may not objectively sleep worse than males in the general population. The likely reason for the finding might be that male resident doctors face more life stress than their female counterparts during the standardized residency training program in the hospital. In addition, some male resident doctors spend more night-time playing games to relax when they feel stressful and thus the reduced sleep time led to poor sleep quality and daily functioning (52). Moreover, in the present study, third-year resident doctors appeared to have a higher insomnia rate than the rest of the counterparts. This could be due to the senior residence status, accompanied by heavier burdens of studies, clinical tasks, and examinations.

The present study not only identified moderating role of resilience in the association between workplace and subjective wellbeing among Chinese resident doctors, but also found the mediating effects of insomnia severity and resilience between them. Although a previous study has provided evidence that workplace bullying increases insomnia, resulting in reduced life satisfaction of employee (18), there was still a paucity of research evaluating the mediating and moderating variables in the association between workplace bullying and subjective wellbeing.

Some studies reported different kinds of bullying and their adverse effects on subjective health complaints or life satisfaction (18, 53). A study conducted in 2017 reported a close relationship between workplace bullying and subjective wellbeing, with personality playing a moderating effect between them (54). In 2019, the data from the second wave of the Children's Worlds Survey revealed that the combined impact of physical and psychological bullying significantly contributed to subjective wellbeing across different age groups and geographical regions (55). Until 2020, another relevant study revealed the moderating effect of resilience on the relationship between poly-bullying victimization and subjective wellbeing (56).

The roles of insomnia severity and resilience in the association between workplace bullying and subjective wellbeing provide an in-depth understanding of the mechanisms. Insomnia severity played a mediating role whereas resilience played both moderating and mediating roles. The current study indicated that resilience may be pivotal among workplace bullying, insomnia severity, and subjective wellbeing. On the other hand, insomnia severity acts as a critical mediator between workplace bullying and subjective wellbeing. Therefore, great resilience is a virtuous circle. According to resilience framework theory, people with positive psychological resilience can promote the reintegration of resilience, hence enabling the individuals to subsequently reach greater resilience (57). Further, resilience is an important component of psychological capital, which is considered as a stable and lasting predictor for subjective wellbeing of an individual (58). Therefore, improving the workplace environment is the first measure that needs to be adopted in the healthcare sector. It is hence important to enhance resilience of resident doctors and reduce their insomnia severity.

Strengths in the present study include large sample size and examination of multiple psychological variables, which provide an in-depth understanding of associations among workplace bullying, resilience, insomnia severity, and subjective wellbeing. In addition, some limitations should be considered in future studies. For example, the present cross-sectional study could not clearly confirm the causality between the variables. In addition, the results in the present study cannot be extrapolated to those with mental disorders.

## Conclusion

The current study found a remarkably high rate of workplace bullying accompanied by a high prevalence of insomnia among the Chinese resident doctors. Further, workplace bullying and poor resilience were the factors associated with insomnia. The present study further validated the mediating effect of insomnia severity on the association between workplace bullying and subjective wellbeing, as well as the moderating and mediating effects of resilience between them. Therefore, resilience may be pivotal in workplace bullying, insomnia severity and subjective wellbeing. Further, insomnia severity acts as a critical mediator between workplace bullying and subjective wellbeing.

## Data Availability Statement

The raw data supporting the conclusions of this article will be made available by the authors, without undue reservation.

## Author Contributions

SZ and JC contributed to conception and design of the study. SZ, JC, and HL organized the research and collected the data. SZ, JC, and YY performed the statistical analysis. JC wrote the first draft of the manuscript. SZ and HL wrote sections of the manuscript. All authors carried out the research. All authors contributed to manuscript revision, read, and approved the submitted version.

## Conflict of Interest

The authors declare that the research was conducted in the absence of any commercial or financial relationships that could be construed as a potential conflict of interest.

## Publisher's Note

All claims expressed in this article are solely those of the authors and do not necessarily represent those of their affiliated organizations, or those of the publisher, the editors and the reviewers. Any product that may be evaluated in this article, or claim that may be made by its manufacturer, is not guaranteed or endorsed by the publisher.
